# Effect of Hydrolysate Derived from Subcritical Seawater Treatment of Buckwheat Waste on the Growth of Lettuce (*Lactuca sativa* L.)

**DOI:** 10.3390/plants14020149

**Published:** 2025-01-07

**Authors:** Yongheng Yuan, Faqinwei Li, Naoto Shimizu

**Affiliations:** 1 Institute of Modern Agricultural Equipment, Xihua University, Chengdu 610039, China; yyh@xhu.edu.cn (Y.Y.); lfqw@xhu.edu.cn (F.L.); 2 Research Faculty of Agriculture, Hokkaido University, Sapporo 060-8589, Japan

**Keywords:** subcritical seawater hydrolysis, buckwheat waste, organic components, nutrient elements, lettuce (*Lactuca sativa* L.)

## Abstract

This study explores the effects of a subcritical seawater treatment (SST) on buckwheat waste (BW), and the use of the hydrolysate as a liquid fertilizer to improve the growth of lettuce (*Lactuca sativa* L.). Three temperature treatments (110 °C, 170 °C, 230 °C) were used for the SST, and the ionic composition in the seawater achieved the depolymerization and degradation of BW. The X-ray diffraction of the residual solids showed that the structure of BW was destroyed. Compared with seawater, the hydrolysate contained higher amounts of elements beneficial to plant growth, such as N, P, K, and organic compounds such as phenolics and sugars, as a result of the degradation of BW caused by the SST. The hydrolysate was tested as a liquid fertilizer (treatments H_110°C_, H_170°C_, H_230°C_) to irrigate lettuce. The content of proteins, phenolics, and chlorophyll, as well as the weight of the lettuce in the H_110°C_ and H_170°C_ treatments, were significantly higher than those in the seawater and the H_230°C_ irrigation treatments (*p* < 0.05). The hydrolysate from the SST of BW, being rich in various organic and inorganic nutrients, can act as a liquid fertilizer that promotes the growth of lettuce, whereas hydrolysate from higher SST temperatures might inhibit the growth of lettuce, because of the excessive total nitrogen and organic acid.

## 1. Introduction

Buckwheat cultivation has increased world-wide because of its increasing popularity as a healthy food [1]. It has been planted for food and medicine, due to the amino acid composition and its contents of fiber, resistant starch, trace elements, vitamins, and antioxidants [2,3,4]. Increased cultivation has led to the production of larger quantities of buckwheat waste (BW) which includes husks, leaves, and stalks. It is usually burned, dumped, or left unused, which has resulted in pollution. BW is rich in cellulose, hemicellulose, antioxidants, and various sugars [5,6,7]; thus, it can be considered as a potential lignocellulose biomass source for improving the economic benefits of buckwheat cultivation.

In recent years, research into the industrial uses of common lignocellulosic biomass has been focused on (1) promoting the degradation of lignocellulosic biomass to extract liquid biofuels [8]; (2) breaking down the physical structure of the biomass followed by enzymatic degradation or fermentation [9]; and (3) creating modified biochar [10]. However, the structure of such biomass is robust and so, despite the fact that these treatments often involve a significant amount of chemical reagents or energy consumption in the early stages, the yield of the target products obtained in the later stages is liable to be unsatisfactory, leading to a situation where the costs outweigh the benefits [11,12]. Therefore, as well as focusing research on the preliminary treatment of lignocellulose biomass, it is also crucial to explore a more rational and effective utilization pathway for the products, in order to achieve a method that is low in input, high in efficiency, and more environmentally friendly.

During the subcritical liquid treatment, a solution is heated above its critical point but below the supercritical point, using high temperature and pressure to generate H^+^ and OH^−^ ions, which break inter- and intra-molecular chemical bonds, degrade the structure of lignocellulosic biomass, and promote the rapid progress of the reaction [13]. This method has become one of the widely used ways to process biomass, because of its rapid reaction and low resulting pollution [14]. However, the process requires a significant consumption of fresh water resources, which are scarce, especially in certain countries and regions. This disadvantage hinders the industrial use of lignocellulosic biomass [15].

Ninety-seven percent of the Earth’s water resources are seawater, which is abundant and inexpensive. Seawater contains ions such as K^+^, Ca^2+^, and Mg^2+^, which are essential inorganic nutrients for plant growth. Despite the high concentration of Na^+^ in seawater, it is considered a beneficial nutrient for plants, and in some cases, it can even replace some of the functions of K^+^ [16]. When seawater is used for subcritical treatment of lignocellulosic biomass, the metal ions in seawater provide an indirect weak acidity that degrades hemicellulose into small oligosaccharides, while the Cl^−^ ions can break the hydrogen bonds in polysaccharides, enhancing the degradation of polysaccharides into monosaccharides [17]. These oligo- and polysaccharides are beneficial for improving soil properties and enhancing plant growth and defense mechanisms against pathogen infections [18]. The subcritical seawater treatment (SST) to degrade lignocellulosic biomass can also produce organic acids (formic acid and acetic acid), amino acids, and phenolic compounds [8], which act as biostimulants and plant growth regulators that promote plant growth [19,20,21].

Lettuce (*Lactuca sativa* L.) is one of the most economically valuable leafy vegetable crops grown worldwide. Lettuce is rich in a variety of nutrients and healthful compounds such as minerals, proteins, and carbohydrates [22], and brings a pleasant flavor to salads and other dishes. In addition, lettuce contains several antioxidants such as ascorbic acid, phenols, and carotenoids [23]. Therefore, the regular consumption of lettuce is very beneficial to human health. Meanwhile, the growth and quality of lettuce are affected by the type of fertilization [24].

In this study, different treatment temperatures (110 °C, 170 °C, 230 °C) were used for the SST of buckwheat waste. The crystallinity of the residual solid and the physicochemical properties of the hydrolysate were analyzed. The collected hydrolysate was diluted and used as a liquid fertilizer (with treatments H_110°C_, H_170°C_, H_230°C_) for the cultivation of lettuce. The growth indicators and physicochemical properties of lettuce grown using the three hydrolysates were compared with those of lettuce grown in seawater. This study explores a route for the use of lignocellulosic biomass, improves the high water resource consumption during subcritical liquid treatments, and offers a reference for the application of seawater in agricultural cultivation.

## 2. Results

### 2.1. Crystallinity of Solid Samples

**Figure 1:**
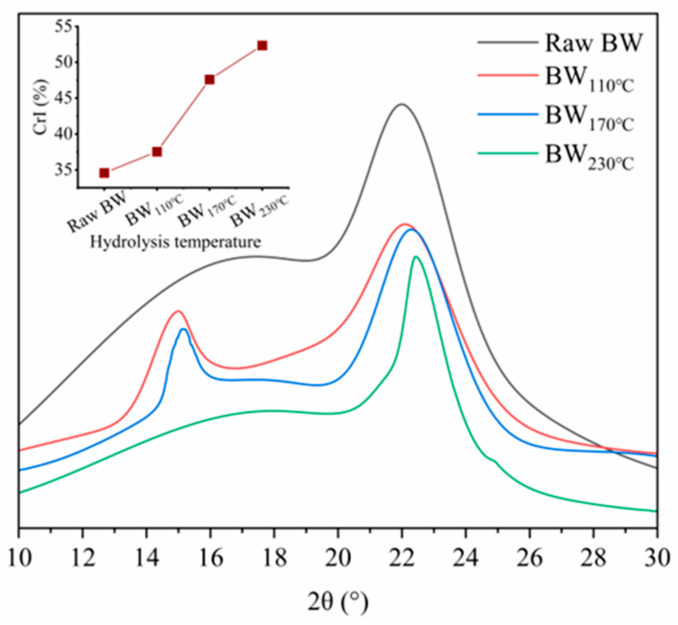
X-ray diffraction patterns and crystallinity index of buckwheat waste after subcritical seawater treatment at different temperatures. (CrI: crystallinity index, BW: buckwheat waste).

An XRD analysis was conducted to determine the crystallinity of raw BW and all samples and to calculate the corresponding crystallinity index (CrI) values (Figure 1). As the treatment temperature increased, the CrI values also increased, from around 35% for raw BW (control group) to 52% after the SST at 230 °C. The increase in CrI indicated that the high-temperature SST of BW led to the disruption of the crystal structure, with the effective removal or degradation of components such as lignin and xylan.

### 2.2. Yields of Organic Components in Hydrolysate

**Figure 2:**
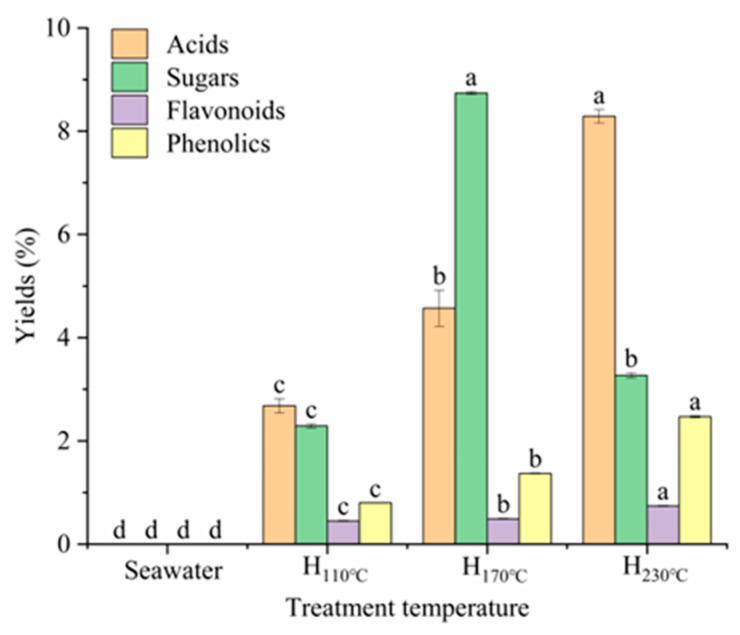
Organic components in the hydrolysate of buckwheat waste after different temperature treatments. Different letters indicate significant differences between the group (*p* < 0.05) based on Duncan’s multiple range test. Values represent means ± SE (n = 3) for individual sample. (H: hydrolysate).

During the SST, the BW undergoes the degradation and dissolution of free components to produce organic compounds. These organic compounds typically include sugars, flavonoids, phenolics, and acidic components. Their contents in BW hydrolysates were measured and are shown in Figure 2. As can be seen from the assay results, the amount of these components in seawater is so small that it is neglected. The yields of phenolic and flavonoid components reached 0.8 ± 0.00% and 0.45 ± 0.01%, respectively, during the 110 °C treatment. The contents of these two substances increased significantly (*p* < 0.05) with increasing temperature, reaching maximum values of 2.47 ± 0.02% and 0.74 ± 0.01% yields, respectively, with the treatment at 230 °C. The content of sugars in the hydrolysates is 2.29 ± 0.04% with the treatment at 110 °C. There was a noticeable increase (*p* < 0.05) from the 110 °C to 170 °C treatment (Figure 2). The maximum yield of 8.74 ± 0.03% was obtained at 170 °C (Figure 2). However, at the highest temperature (230 °C) tested, the content of sugars showed a marked decrease compared with that produced at 170 °C (*p* < 0.05). The sugar yield reduced to 3.27 ± 0.05%. The content of acids in the hydrolysate also increased with higher-temperature treatments, rising from 2.68 ± 0.14% at 110 °C to the maximum yield of 8.29 ± 0.13% produced at 230 °C (Figure 2).

### 2.3. Nutritional Elements in Hydrolysates

The quantities of certain elements detected in the hydrolysate are shown in Table 1. These elements are typically the main components of seawater and are also essential elements required for plant growth. Compared with seawater, the contents of all elements measured in the hydrolysate (except Ca^2+^) were higher and most of them increased with higher treatment temperatures. For instance, the total N content in the hydrolysate was 10 mg/L in seawater and increased with a higher treatment temperature to 200.03 mg/L at 230 °C. The content of K^+^ showed an increasing and then decreasing trend after the subcritical water treatment. The K^+^ content in seawater was 182.49 mg/L, and the maximum value of 597.43 mg/L was obtained at 110 °C, and then decreased to 589.11 mg/L with the increase in temperature. The Mg^2+^ content was detected to be the highest in the hydrolysate, reaching 749.95 mg/L after treatment at 230 °C from 612.05 mg/L in seawater, whereas the Ca^2+^ content decreased, from 199.96 mg/L in seawater to only 100.03 mg/L after treatment at 230 °C.

### 2.4. Liquid Components of Hydrolysates and Solid Residues

The volume of the supernatant liquid and its electrical conductivity (EC) and pH were measured (Table 2). The EC value of seawater is quite low; after the subcritical water treatment at each temperature, the EC values of the hydrolysates were slightly higher than seawater (*p* < 0.05), with the maximum EC value of 22.60 ± 0.31 mS/cm obtained at 230 °C. The pH value was 7.23 ± 0.09 in the seawater but was lower in each hydrolysate, with the minimum pH value of 3.29 ± 0.03 obtained at 230 °C, consistent with the supposition. The pH values decreased with the increase in temperature, corresponding to the progressive increase in the obtained acid content. The volume of the hydrolysate was also measured. The amount of seawater used was 150 mL added to 6 g of BW. After the subcritical treatment, the volumes of the hydrolysate at 110 °C and 170 °C were 106.33 ± 2.52 mL and 106.00 ± 3.61 mL, respectively, and at 230 °C, the volume was 116.67 ± 3.06 mL. The weight of the dried solid residue after centrifuging was also measured; like Table 3 shows, as the temperature increased, the weight of the residual solids decreased, with the minimum value of 3.29 ± 0.22 g of residue obtained at 230 °C.

### 2.5. Quality Parameters and SPAD in Lettuce

**Figure 3:**
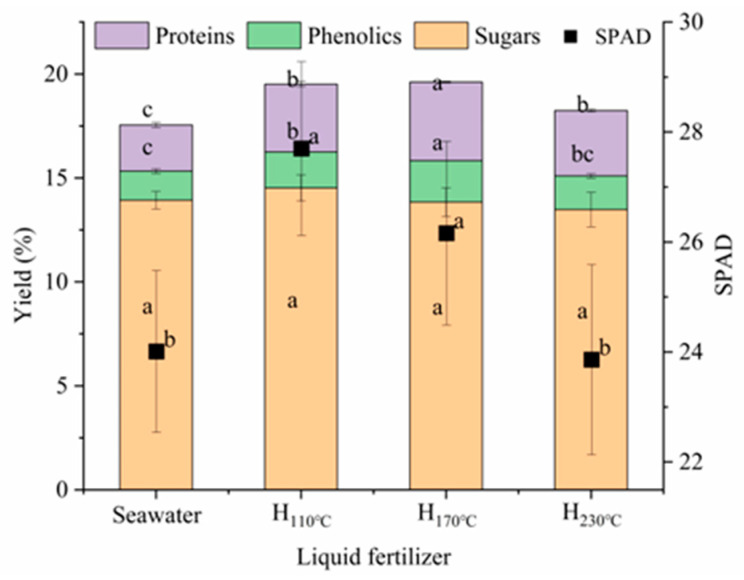
Content of proteins, phenolics, and sugars, and SPAD values of lettuce. Different letters indicate significant differences between the group (*p* < 0.05) based on Duncan’s multiple range test. Values represent means ± SE (n = 3) for individual sample. (H: hydrolysate).

Figure 3 presents the content of proteins, phenolic compounds, and sugars in lettuce, as well as the results of the SPAD detection as an indication of the chlorophyll content. The content of proteins and phenolic compounds showed similar trends, increasing and then decreasing with the treatment temperature, relative to the control plants, and reaching maximum values of 3.78 ± 0.04% and 1.99 ± 0.01%, respectively, in the H_170°C_ treatment, with significant differences (*p* < 0.05). As a chlorophyll content index, the SPAD value was measured. It also showed an increasing and then decreasing trend; the chlorophyll contents of lettuce from the H_110°C_ and H_170°C_ groups were significantly higher than control plants and H_230°C_ groups, with the maximum value of 27.70 ± 1.58 obtained in H_110°C_. However, there was no significant difference (*p* > 0.05) between H_110°C_ and H_170°C_.

### 2.6. Growth Parameters in Lettuce

**Figure 4:**
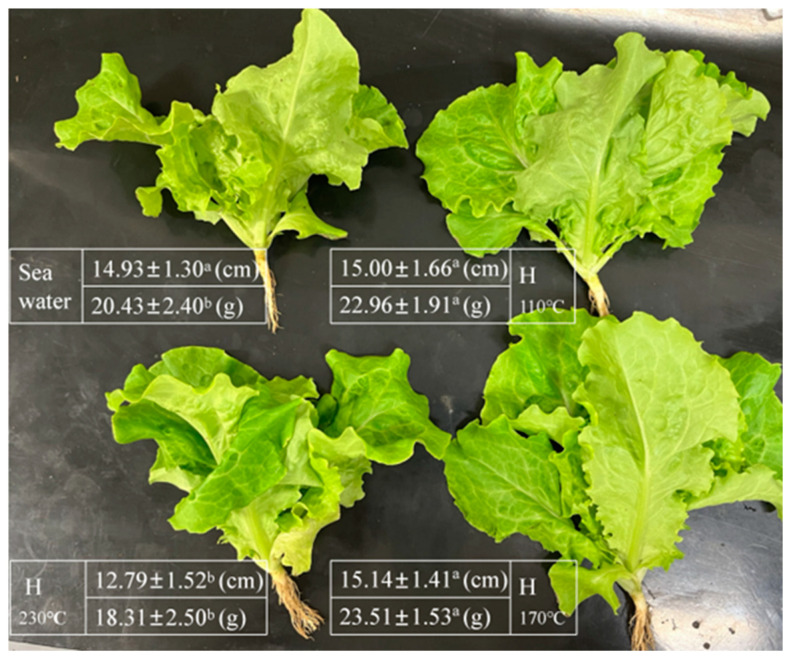
Height and weight of lettuce in different hydrolysate treatment groups. Different letters indicate significant differences between the group (*p* < 0.05) based on Duncan’s multiple range test. Values represent means ± SE (n = 7) for individual sample. (H: hydrolysate).

Figure 4 illustrates the height and weight of lettuce, with all crops harvested after 18 d of growth. The results show that lettuce irrigated with hydrolysate did not exhibit significant changes in height (*p* > 0.05). However, the yield (weight) of lettuce showed an increasing and then decreasing trend, with significantly higher weights in H_110°C_ and H_170°C_ groups (*p* < 0.05) compared with the control plant and H_230°C_, and a maximum value of 23.51 ± 1.53 g in H_170°C_, but no significant difference (*p* > 0.05) between H_110°C_ and H_170°C_.

## 3. Discussion

### 3.1. Effects of Organic Components in Hydrolysate

**Figure 5:**
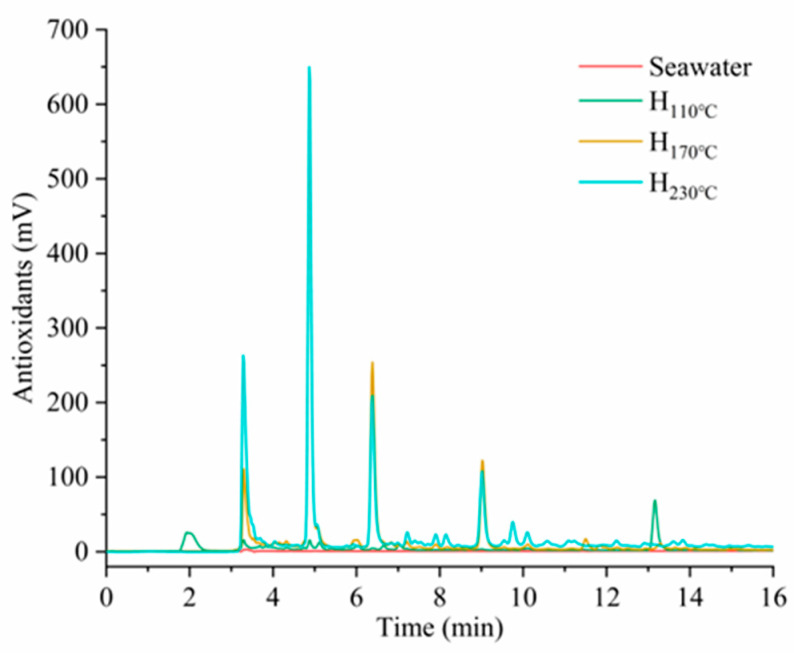
HPLC analysis of antioxidant substances in hydrolysates. (H: hydrolysate).

The total phenolics and flavonoids in the hydrolysate would originate from the dissolution of free components in the BW and the degradation of lignin at high temperatures [25]. This result indicated that an increase in temperature promoted the degradation of lignin in the BW and the dissolution of these substances. Previous studies have shown that plant biomass will degrade during hydrothermal treatment in seawater and dissolve into amino acids, phenolics, and sugars; the salts in seawater promote the formation of acids during the degradation process [8]. For antioxidant compounds, Figure 5 shows that, after hydrolysis at different temperatures, the area of the peaks for each substance varied independently, indicating that these components might undergo different extents of degradation or transformation at different temperatures.

The increase in sugar is attributed to a report that inorganic salts (NaCl, MgSO_4_, CaCl_2_, FeCl_3_, FeSO_4_, Fe_2_(SO_4_)_3_) play a significant role in the degradation of xylan in some agricultural waste into xylose and even glucose. The metal ions in these inorganic salts provide indirect weak acidity (Lewis acid) and, at the same time, a small amount of acetic acid is produced during the hydrothermal process, which might promote the degradation of hemicellulose into smaller oligosaccharides [17]. In addition, some studies have shown that, under certain temperatures and acidic conditions, chloride ions break the hydrogen bonds between polysaccharide molecules, thus promoting the degradation of high-molecular-weight polysaccharides into smaller polysaccharides or monosaccharides [26,27]. The seawater treatment can combine these effects to form a composite degradation system, which better promotes the depolymerization of hemicellulose under mild temperatures and pressures. However, the decrease in the sugar yield under 230 °C treatment may be due to the further degradation of sugar at high temperatures. This finding was similar to the results of Zhang et al. [17] and is considered to be the case because higher temperatures destroy the structure of xylan; coupled with the catalytic action of the composite system, higher temperatures lead to the further degradation of xylooligosaccharides into xylose and furfural.

**Figure 6:**
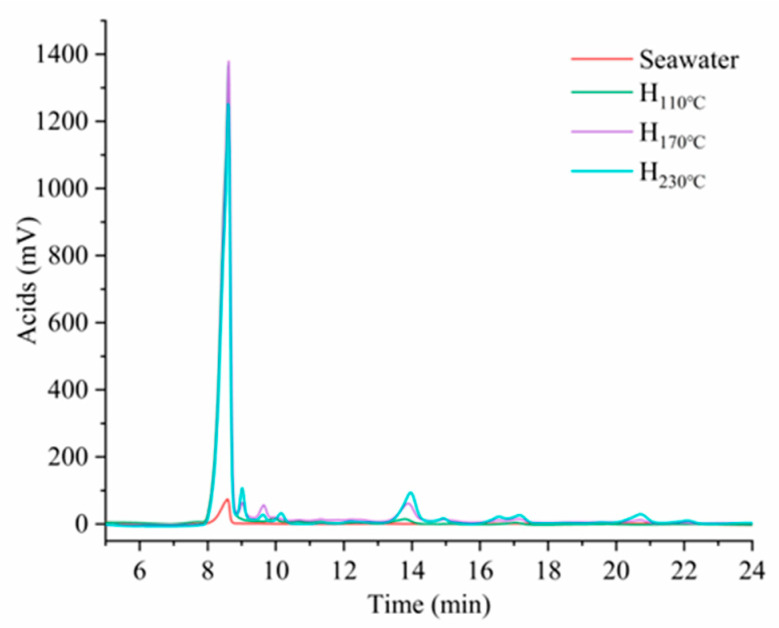
HPLC analysis of acidic substances in the hydrolysates produced at different treatment temperatures. (H: hydrolysate).

These acids might originate from the dissolution of volatile acids inherent in BW, and might also arise from the degradation of sugars, such as the small amount of acetic acid produced during the hydrothermal process, as mentioned earlier [17]. Glucuronic acid and acetic acid can also be derived from the decomposition of glucuronic and o-acetyl groups in the side chains of hemicellulose [28]. Low-molecular-weight organic acids, such as glycolic acid and formic acid, are further degradation products [29]. Yu et al. mentioned that after treatment with FeCl_2_, a large amount of hemicellulose was degraded, and most of the sugars produced were further degraded into acids. Salts of Zn^2+^ or Co^2+^ can increase the formation of lactic acid [29], from the ability of Lewis acids to convert trioses, such as glyceraldehyde and dihydroxyacetone, into lactic acid [30,31]. Seawater salts at 200 °C promoted the formation of carboxylic acids (formic acid and acetic acid) during degradation [8]. In addition, some phenolic components also contribute to acidity, such as gallic acid [25]. Such acidic components might also degrade further or transform with changes in temperature. For example, Figure 6 shows that the peak of acidic components at around 8 min was the highest in the hydrolysate produced at 170 °C, yet the peak at around 14 min was the highest in that at 230 °C.

### 3.2. Effects of Nutritional Elements in the Hydrolysate

Elevated levels of these elements (N, P, K, Mg^2+^, Mn^2+^, Zn^2+^) are likely a result of the degradation of BW. For example, the rise in K^+^ after the subcritical seawater treatment might occur because plants naturally contain a large amount of K^+^ (for example, in plant vacuoles). When BW is treated with the SST, its structure is disrupted, and as the temperature rises, the degree of degradation of the lignocellulose cell wall increases, leading to the degradation of vacuoles and the release of K^+^. Therefore, the content of K^+^ in the hydrolysate would be expected to increase. However, dissolved K^+^ is able to form complexes with polysaccharides [32], which can easily adsorb onto the fiber surface, resulting in a reduced proportion of K^+^ dissolved in the hydrolysate after higher-temperature treatments (170 °C and 230 °C); this was similar to the results of Ge et al. [33]. Mg^2+^ exists in unstable compounds within lignocellulose, is an important component of polyribosomes, and can promote the synthesis of vitamins in crops [34]. Mg^2+^ within cells can be free in the cytoplasm or attached to the surface of the endoplasmic reticulum. As the temperature rises, the outer layer of the cell wall is destroyed and Mg^2+^ is rapidly released from the cytoplasm, leading to an increase in the concentration of Mg^2+^ in the hydrolysate. With a continued rise in temperature, proteins, lipids, and sugars are dissociated and dissolved in the hydrolysate, releasing Mg^2+^ that is bound to these components, thus further increasing the dissolution of Mg^2+^ [33]. The content of Ca^2+^ in the hydrolysate, in contrast, decreased with the increase in the treatment temperature. It is known that OH^−^ and oxalate (C_2_O_4_^2−^) ions are produced during the subcritical seawater treatment of biomass; so, the decrease in Ca^2+^ might be attributed to the formation of precipitates such as calcium oxalate.

### 3.3. Effects of Liquid Components in the Hydrolysates and Solid Residues

The increase in the EC value might be attributed to two factors. On one hand, from the analysis of the elemental content of the hydrolysates (Table 1), it can be surmised that the SST caused some ions from BW to dissolve into the hydrolysate, thereby increasing its EC value. On the other hand, the increase in the EC value might also be related to the acids produced by the degradation of BW: those acids would ionize to produce H^+^, and the increase in ions would also lead to an increase in the EC value. The obtained volume of the hydrolysate was less than the original amount added because, during the SST process, some seawater permeates and is retained in the BW by the action of forces or chemical bonds. It is noteworthy that when the treatment temperature was 230 °C, the volume of the hydrolysate was higher than those at 110 °C and 170 °C. This might be caused by the greater degradation of BW at the higher temperature, leading to a reduction in the amount of solids and consequently a decrease in the amount of liquid attached to the solids. The weight of residual solids in each treatment also supports this view: as the temperature increased, the weight of the residual solids decreased. This result further indicates that, under the SST, BW is effectively degraded, with a substantial amount of BW components dissolved into the hydrolysate.

### 3.4. Effects of Hydrolysate Application on Lettuce

The protein and phenolic compound results indicate that the organic substances contained in the hydrolysate after the SST effectively increased the nutritional content in lettuce. The elements N, P, K, Ca, Mg, etc., contained in the hydrolysate are essential macronutrients for plant growth. In addition, the hydrolysates were rich in trace elements (Mn, Zn, etc.), which are also crucial for plant growth. The organic content obtained from the SST hydrolysates of BW also promotes plant growth. For instance, polysaccharides extracted from BW are likely to be beneficial for improving soil properties and enhancing plant growth and defense mechanisms against pathogen infections. Polysaccharides are stable over a wide range of pH levels and temperatures and combine with water to produce highly viscous solutions. Moreover, polysaccharides increase the soil water retention capacity, thereby improving soil health compared with raw soil [18]. At the same time, the phenolic and flavonoid compounds in the hydrolysate, in addition to their antioxidant capabilities, possess antifungal properties [35,36]. The antifungal mechanism of phenolics is based on the inactivation of fungal enzymes that contain thiol groups in their active sites [37,38,39]. Hassan et al. and Khoulati et al. also reported similar results: Hassan et al. found that various plant hormones, flavonoids, amino acids, antioxidants, and essential nutrients extracted from moringa leaf can act as biostimulants and plant growth promoters [19,20,21]. Khoulati et al. [40] reported that the complex composition of saffron extractives improved the growth of tomatoes, citing nitrogenous substances, glycosides, aldehydes, flavonoids, volatile oils, proteins, carbohydrates [41], Na, K, Zn, P, Mn, Mg, Ca, and Fe [42], and some amino acids [43]. In addition, Li et al. [28] reported a strong correlation between SPAD and total N, indicating that the increase in N content in the SST hydrolysate of BW might be one of the reasons for the increased chlorophyll content in lettuce. However, in H_230°C_, the chlorophyll content was lower than that in the other treatments, suggesting that if the total N content were too high, it might affect plant photosynthesis, leading to a reduction in chlorophyll content. There also was a lower content of organic components in the plants in H_230°C_. This might be caused by several factors, aside from the total N effect. On the one hand, it is possible that the high content of certain organic components (such as acids) in the hydrolysate might disrupt the nutritional balance in the soil, thereby affecting the plants’ absorption of nutrients from the soil and so inhibiting the growth of lettuce. On the other hand, it is also possible that the higher-temperature SST degradation produced substances harmful to plant growth, or lead to a decrease in the activity of beneficial soil microorganisms, thus affecting plant growth. These possibilities require further experimental investigation in the future. In addition, it is worth noting that the content of soluble sugars in the lettuce did not show any significant changes with the hydrolysate treatment (*p* > 0.05), and the reasons for this also need further exploration and analysis. A substantial amount of research has shown that applying too much N fertilizer can reduce crop yields: the excessive application of N can lead to soil acidification, thereby affecting plant growth [44]. The results of weight confirmed that the application of the hydrolysate had a growth-promoting effect on lettuce. An appropriate NO_3_^−^/NH_4_^+^ ratio promotes an increase in the plant yield [45]. It can be surmised that lettuce, as a nitrate-loving crop [46], accumulated biomass because of the increased nitrate-N in the hydrolysate. However, there was no significant difference in the weight of the hydrolysate-irrigated lettuce obtained in the 110 °C and 170 °C groups (*p* > 0.05). It is well-recognized that high temperatures also lead to increased energy consumption during subcritical processing. Therefore, the hydrolysate obtained during the treatment at 110 °C may be the most suitable.

## 4. Materials and Methods

### 4.1. Subcritical Seawater Treatment

Buckwheat waste (BW) was obtained from the experimental fields of Hokkaido University, and the seawater was sourced from coordinates 43°9′ N, 141°12′ E. Seawater is vacuum-evacuated with a 0.45um membrane before use to remove sand, small particles, and other impurities in the seawater. The treatment was conducted in a 200 mL reaction vessel, in which 6 g BW was mixed with 150 mL seawater and reacted at 110 °C, 170 °C, or 230 °C for 45 min, with a rotation speed of 800 rpm during the reaction. The operating pressure changed with increases in the temperature and maintenance period. The reaction vessel was then placed in cold water to cool to room temperature. Subsequently, the mixture was centrifuged at 8000 rpm for 20 min to separate the solid from the liquid. The volume of the supernatant liquid was recorded using a measuring cylinder and the liquid was stored at 4 °C until further use. All the chemical reagents used are purchased from Wako Pure Chemical Industries, Ltd., located in Osaka, Japan; the residual solids were also collected and dried at 50 °C to constant weight.

### 4.2. Crystallinity of Buckwheat Waste

The crystallinity of BW in the solid samples was determined using X-ray diffraction (XRD). The scanning range was set from 5° to 80°, with a scanning speed of 4° per minute and a step size of 0.02°. An X-ray diffractometer (LabX XRD-6100, Shimadzu, Kyoto, Japan) was used, operating at 40 kV (λ = 0.154 nm) to analyze the crystallinity. The CrI was calculated using the following equation by Segal et al. [47]:

$$\mathrm{CrI}\;(\%)=\frac{I_{002}-I_{am}}{I_{002}}\times 100$$

where I_002_ is the diffraction intensity of crystalline structure at 2θ of 22.5° and I_am_ is the diffraction intensity of the amorphous portion at 2θ of 18°.

### 4.3. Content of Sugars in the Hydrolysate

The content of sugars was determined using the 3,5-dinitrosalicylic acid method [48]. Glucose was used as a reference compound to establish a calibration curve. All samples were analyzed in triplicate.

### 4.4. Content of Phenolics in the Hydrolysate

To determine total phenolic compounds, a sample (0.5 mL) of the liquid hydrolysate was added to 2.5 mL 10% Folin–Ciocalteu (FC) reagent, mixed well, and gently agitated for 10 min. Then, 2.5 mL of 7.5% sodium carbonate (Na_2_CO_3_) was added, and the mixture was left to stand in the dark at 45 °C for 1 h. The blank sample contained 0.5 mL of distilled water, 2.5 mL of 10% FC reagent, and 2.5 mL of 7.5% Na_2_CO_3_. The absorbance at 765 nm was measured using a UV-visible spectrophotometer. A calibration curve was plotted using gallic acid as a reference compound. All samples were analyzed in triplicate.

### 4.5. Content of Flavonoids in the Hydrolysate

To determine total flavonoid compounds, 1 mL of the sample was diluted with distilled water 5 times then mixed with 0.3 mL of a 5% NaNO_2_ solution. The mixture was allowed to react for 6 min; then, 0.3 mL of 10% AlCl_3_ solution was added and allowed to react for 5 min. Finally, 2 mL of a 1 mol/L NaOH solution was added. After the reaction for 15 min, the absorbance at 510 nm was measured using a UV-visible spectrophotometer. The liquid sample was replaced with distilled water as a blank control. Rutin was used as a reference compound to establish a calibration curve. All samples were analyzed in triplicate.

### 4.6. Content of Total Acids in the Hydrolysate

To determine total acids, 5 mL of the sample diluted with distilled water 5 times was added to 15 mL of water and 100 μL of the phenolphthalein indicator. The mixture was titrated with 0.1 mol/L of NaOH until the solution first showed a pink color and did not change color within 0.5 min. The amount of NaOH solution used was recorded. The sample was replaced with distilled water for titration as a blank control. All samples were analyzed in triplicate.

### 4.7. HPLC Analysis of Organic Acids and Antioxidants

The content of organic acids in the hydrolysate was determined by high-performance liquid chromatography (HPLC) using an Agilent 1260 series instrument (Agilent Technologies, Santa Clara, CA, USA), equipped with an RSpak KC-811 chromatographic column with a KC-G guard column (Shodex, Tokyo, Japan), and a UV detector set at 210 nm [25]. The mobile phase was 1 mol/L of HClO_4_, with a flow rate of 0.7 mL/min and an injection volume of 50 μL.

The content of antioxidants in the hydrolysate was determined by HPLC equipped with a UV detector and a C18M E chromatographic column, with the column temperature set at 35 °C [25]. The mobile phase consisted of 100% methanol (solvent A) and 0.5% acetic acid aqueous solution (solvent B), with a gradient elution of 30–90% of solvent A and 70–10% of solvent B over 25 min, at a flow rate of 0.8 mL/min. An injection volume of 10 µL was used, and the absorption of each component was detected at 290 nm.

### 4.8. Hydrolysate pH and Electrical Conductivity, and Content of Nutrient Elements in Lettuce

The pH and electrical conductivity of the hydrolysate were determined using a WD-35634-30 digital pH meter (Oakton Instruments, Vernon Hills, IL, USA) and EC meter (D-220PC-S HI9813-5N, Hanna Instruments, Chiba, Japan), respectively [49]. The nutrient elements in lettuce were measured by the Tokachi Agricultural Cooperative Federation, Agricultural Chemical Research Institute. The total nitrogen, ammonium nitrogen, and nitrate nitrogen contents in hydrolysate were determined in accordance with method of Lu et al. [50]. The liquid sample was dried firstly, and then approximately 50 mg of the residue solid was added to 2 mL of 61% HNO_3_ (EL grade; Kanto Chemical, Tokyo, Japan) in a test tube and heated at 110 °C in a DigiPREP MS apparatus (SCP Science, Québec, QC, Canada) until the powder had almost disappeared. Then, 0.5 mL of H_2_O_2_ (semiconductor grade; Santoku Chemical, Tokyo, Japan) was added twice and continually heated until the solution became limpid. After cooling, the test tube was filled to 10 mL with 2% HNO_3_ and analyzed by ICP-MS (Elan, DRC-e; PerkinElmer, Waltham, MA, USA) for the following elements: P, K, Ca, Mg, Mn, and Zn. The compound multielement standard solution IV (Merck, Tokyo, Japan) was used for ionomic determination [51].

### 4.9. Cultivation of Lettuce

Lettuce seeds (cv. Grand Rapids) were purchased from Sakata Seed Corporation Ltd. (Yokohama, Japan). The experiments were conducted in a plastic film greenhouse on the campus of Hokkaido University (43°4′ N, 141°20′ E; altitude 20 m). The seeds were washed and soaked in warm water at 55 °C for 5 min, then wrapped in damp cheesecloth to promote germination at 25 °C for 48 h. After germination, the seeds were sown on 12 April 2023 into a seedling tray with 40 compartments (one seed per compartment). Commercial nutrition soils (Iris Ohyama Corporation Ltd., Sendai, Japan) were used as the seedling medium. After 20 d, uniformly healthy seedlings were transplanted into the experimental pot (Height: 16.3 cm, Diameter: 13.2 cm). Thereafter, the seedlings were watered with the hydrolysate produced by the SST under different temperature conditions, while the seedlings in the control group were watered with seawater, which was diluted with the same concentrations. The pot experiment was conducted using a completely randomized factorial design, with each group of experiments repeated with seven lettuce plants. The hydrolysate was diluted with distilled water to a concentration of 8.33% (10 mL hydrolysate mixed with 1190 mL distilled water) as the liquid fertilizer, and 40 mL was watered daily for 18 d, after which the growth indicators of the plants were measured and harvested. The freshly harvested lettuce including leaves and stems was ground, mixed with 25 mL distilled water, filtered, and the filtrate was tested for total phenolics, sugars, and proteins. All samples were analyzed in triplicate.

### 4.10. Content of Proteins, Total Phenolics, and Sugars in Lettuce Extracts

The detection of proteins was performed using the Coomassie Brilliant Blue method: 1 mL of lettuce extract diluted with distilled water was added to 5 mL of Coomassie Brilliant Blue, mixed well, reacted for 5 min, and then the absorbance at 595 nm was measured. The detection methods for sugars and total phenolics were the same as those used for the SST hydrolysate of BW (Section 4.3 and Section 4.4). All samples were analyzed in triplicate.

### 4.11. Measurement of Lettuce Height, Weight, and SPAD Value

The SPAD value was used as a chlorophyll content index and was measured before the harvest using a portable chlorophyll meter (SPAD-502, Minolta Camera Co., Ltd., Tokyo, Japan). The height and weight of the lettuce plants were measured immediately after harvest on 20 May 2023. All samples were analyzed in triplicate.

### 4.12. Statistical Analysis

The experimental data are presented as the mean ± standard error of three replicates. An ANOVA was performed using SPSS Statistical Software (version 25.0) to determine the statistical significance at a 95% confidence interval. The figures were prepared using OriginPro 2021 software (OriginLab, Northampton, MA, USA).

## 5. Conclusions

The subcritical seawater treatment (SST) of buckwheat waste (BW) can break down the structure and degrade BW. It can be surmised that the inorganic salt components in seawater promote the destruction of the plant vacuoles and cytoplasm, thereby releasing K^+^ and Mg^2+^ into the hydrolysate. The acidic conditions of the hydrolysate lead to the degradation of proteins into available N, an essential element for plant growth. The increased temperature of the SST results in a reduction of Ca^2+^ content in the hydrolysate because of the generation of OH^−^ and oxalate ions, which cause calcium to form precipitates. Organic components, such as sugars, acids, and phenolic compounds, increased in the hydrolysate with higher temperatures because of the degradation of BW. The synergistic action of these organic substances and inorganic elements, acting together in a liquid fertilizer, can improve soil health, help resist fungi in the soil, and provide necessary nutrients for the growth of lettuce. Such extracts of agricultural wastes have the potential for making a significant contribution to the high-quality growth of lettuce.

## Figures and Tables

**Table 1:** Content of selected elements in the hydrolysate (mg/L). (H: hydrolysate).

	Seawater	H_110°C_	H_170°C_	H_230°C_
NO_3_^−^-N	0.00	44.70	41.85	41.90
NH_4_^+^-N	27.00	31.26	38.80	54.80
Total N	10.00	100.07	100.12	200.03
P	4.37	21.80	26.21	26.19
K	182.49	597.43	589.11	589.18
Ca	199.96	121.37	114.21	100.03
Mg	612.05	726.02	690.03	749.95
Mn	5.71	7.42	7.74	8.38
Zn	2.79	3.45	3.57	4.45

**Table 2:** Electrical conductivity, pH, and volume of liquid hydrolysates. Different letters indicate significant differences between the group (*p* < 0.05) based on Duncan’s multiple range test. Values represent means ± SE (n = 3) for individual sample. (EC: electrical conductivity).

	EC (mS/cm)	pH	Volume (mL)
Seawater	21.54 ± 0.44 b	7.23 ± 0.09 a	150.00 ± 0.00 a
H_110°C_	22.47 ± 0.48 a	4.29 ± 0.03 b	106.33 ± 2.52 c
H_170°C_	22.58 ± 0.38 a	3.44 ± 0.17 c	106.00 ± 3.61 c
H_230°C_	22.60 ± 0.31 a	3.29 ± 0.03 d	116.67 ± 3.06 b

**Table 3:** Weight of solid residue. Different letters indicate significant differences (*p* < 0.05) based on Duncan’s multiple range test. Values represent means ± SE (n = 3) for individual sample. (H: hydrolysate, BW: buckwheat waste).

	Raw BW	H_110°C_	H_170°C_	H_230°C_
Weight (g)	6.00 ± 0.00 a	5.30 ± 0.25 b	4.23 ± 0.10 c	3.29 ± 0.22 d

## Data Availability

All available data are reported in the paper.
